# Transcriptome analysis of the effect of C-C chemokine receptor 5 deficiency on cell response to *Toxoplasma gondii* in brain cells

**DOI:** 10.1186/s12864-019-6076-4

**Published:** 2019-09-11

**Authors:** Kaoru Kobayashi, Kousuke Umeda, Fumiaki Ihara, Sachi Tanaka, Junya Yamagishi, Yutaka Suzuki, Yoshifumi Nishikawa

**Affiliations:** 1 National Research Center for Protozoan Diseases, Obihiro University of Agriculture and Veterinary Medicine, Obihiro, Hokkaido Japan; 2 Division of Animal Science, Department of Agricultural and Life Sciences, Faculty of Agriculture, Shinshu University, Minamiminowa, Nagano, Japan; 3 Research Center for Zoonosis Control, Hokkaido University, Sapporo, Hokkaido Japan; 4 Graduate School of Frontier Science, The University of Tokyo, Kashiwa, Chiba, Japan

**Keywords:** *Toxoplasma gondii*, C-C chemokine receptor 5, Brain, Astrocyte, Microglia, Neuron, Transcriptome

## Abstract

**Background:**

Infection with *Toxoplasma gondii* is thought to damage the brain and be a risk factor for neurological and psychotic disorders. The immune response-participating chemokine system has recently been considered vital for brain cell signaling and neural functioning. Here, we investigated the effect of the deficiency of C-C chemokine receptor 5 (CCR5), which is previously reported to be associated with *T. gondii* infection, on gene expression in the brain during *T. gondii* infection and the relationship between CCR5 and the inflammatory response against *T. gondii* infection in the brain.

**Results:**

We performed a genome-wide comprehensive analysis of brain cells from wild-type and CCR5-deficient mice. Mouse primary brain cells infected with *T. gondii* were subjected to RNA sequencing. The expression levels of some genes, especially in astrocytes and microglia, were altered by CCR5-deficiency during *T. gondii* infection, and the gene ontology and Kyoto Encyclopedia of Genes and Genomes analysis revealed an enhanced immune response in the brain cells. The expression levels of genes which were highly differentially expressed in vitro were also investigated in the mouse brains during the *T. gondii* infections. Among the genes tested, only *Saa3* (serum amyloid A3) showed partly CCR5-dependent upregulation during the acute infection phase. However, analysis of the subacute phase showed that in addition to *Saa3*, *Hmox1* may also contribute to the protection and/or pathology partly via the CCR5 pathway.

**Conclusions:**

Our results indicate that CCR5 is involved in *T. gondii* infection in the brain where it contributes to inflammatory responses and parasite elimination. We suggest that the inflammatory response by glial cells through CCR5 might be associated with neurological injury during *T. gondii* infection to some extent.

**Supplementary information:**

**Supplementary information** accompanies this paper at 10.1186/s12864-019-6076-4.

## Background

*Toxoplasma gondii* is a protozoan apicomplexan parasite that causes the parasitic disease, toxoplasmosis. It can infect all mammals and birds, commonly through contaminated food [1]. The oocysts in feces from the definitive feline host can also be a source of infection in intermediate hosts such as rodents, birds, livestock, and humans. In these hosts, oocysts transform into tachyzoites shortly after ingestion of contaminated soil, water or plant material. Following exposure to the parasite, infection is divided into acute and chronic stages. The acute stage is characterized by replication of the fast replicating forms of the parasite, the tachyzoites. In contrast, bradyzoites in tissue cysts are responsible for establishing chronic latent infections [1].

Contamination of meat with *T. gondii* bradyzoites represents a major source of human infections [2], and a third of the world’s population is reportedly chronically infected with this parasite [1]. Although the prevalence of latent infections can reach 70% in human populations [3], there is currently no treatment that targets bradyzoite-containing tissue cysts. Toxoplasmosis causes severe clinical diseases in immunocompromised individuals and congenital disorders in infants [4]. In immunocompetent humans, toxoplasmosis is generally asymptomatic, but in patients with acquired immune deficiency syndrome (AIDS), their immunocompromised state reactivates the parasites, leading to the development of toxoplasmosis [5]. Uncontrolled parasite replication can cause life-threatening brain damage characterized by brain abscesses and necrotic areas, and toxoplasmic encephalitis is one of the primary causes of death in HIV-infected people [6]. Primary infections with *T. gondii* in pregnant women can also cause hydrocephalus or developmental disorders in the developing fetus [1].

*T. gondii* is capable of infecting most nucleated cells in vitro [7]. However, acute infections stimulate immune responses in the host, and these responses suppress the proliferation of the parasites [8]. The interferon-gamma (IFN-γ)-mediated immune response is central to this parasite-suppressive response. Many pathogens have developed strategies to modulate host nuclear factor-kappa B (NF-κB) activation and the onwards recruitment and activation of immune cells, which results in enhanced survival of the pathogen. Evidence currently exists to support both inhibition and activation of the NF-κB pathway in the host cells by *T. gondii* [9, 10]. The resultant immune attack and stressed condition inactivate *T. gondii* in most parts of the body. However, the parasite can persist in the brain as encysted bradyzoites [5, 8].

The human brain cell population consists mainly of three types: astrocytes, microglia, and neurons. Astrocytes, which has the highest population in the brain, participate in many brain activities such as brain development [11] and neurotransmission [12]. Microglia are crucial for brain injury and disease [13, 14], and neurons are vital in brain function processes such as neurotransmission and neuroendocrine activities [12]. In the brain, the chemokine system participates in neuroinflammatory processes and the development and functioning of the central nervous system (CNS), which includes neuron-glia communication and neuroendocrine activity [15]. These brain cells can also become infected with *T. gondii* tachyzoites [16]. However, a previous in vivo study showed that *T. gondii* is primarily found in neurons, suggesting that neurons are the primary target cells for this parasite [17]. Controversy exists concerning the effect of *T. gondii* on astrocytes, which may serve as parasite proliferation recipients or protective immune response activators within the CNS [18]. Besides their role in triggering anti-parasite immunity in *T. gondii* infections, microglia may act as “Trojan horses” [19].

Previously, our group reported on the upregulation of immune response-associated genes and downregulation of neurological function-related genes in whole-brain gene expression profiling during *T. gondii* infection [20]. The upregulated genes included chemokines and chemokine receptors; specifically, C-C chemokine ligand 5 (*Ccl5*) and *Ccr5*. C-C chemokine receptor 5 (CCR5) has been mainly studied in the context of HIV infection [21]. This chemokine receptor is expressed in memory and effector T-lymphocytes, natural killer cells, monocytes, macrophages and immature dendritic cells. In these cell types, CCR5 regulates chemotaxis and cell activation, including during *T. gondii* infection [21]. In some previous studies, CCR5-deficient (KO) mice showed increased susceptibility to systemic *T. gondii* infections and an increased number of brain cysts, suggesting that CCR5 is required for innate immunity against *T. gondii* and parasite elimination [22–24]. CCR5 is also expressed in brain cells such as neurons, astrocytes, and microglia. Previous studies on non-infective CNS injury have suggested that CCR5 plays neuroprotective functions [25, 26]. CCR5 is also implicated in the neural function of nociception [27]. A previous clinical study also showed that people with CCR5 chemokine receptor gene polymorphisms have a greater risk of developing ocular toxoplasmosis maybe due to a strong and persistent inflammatory response in ocular tissue. However, a detailed association between the mechanism underlying the inflammatory response during *T. gondii* infection and the role of CCR5 in the brain remains unclear.

Here, we aimed to examine the effect of CCR5-deficiency on gene expression in the brain during *T. gondii* infection and to know the relationship between CCR5 and the inflammatory response against *T. gondii* infection in the brain. We profiled the gene expression patterns of primary brain cells exposed to *T. gondii* tachyzoites using RNA sequencing (RNA-seq). In vivo assays were then used to quantify the expression levels of the genes that appeared to be involved in the control of parasite proliferation and pathology in the mouse brain. We suggest that the inflammatory response through CCR5 might be associated with neurological injury during *T. gondii* infection to some extent.

## Results

### Gene expression in astrocytes during *T. gondii* infection was affected by CCR5-deficiency

Primary astrocytes from wild-type (WT) and CCR5KO mice were infected with *T. gondii* tachyzoites and subjected to RNA-seq followed by differential expression analysis. The analysis identified 115 genes that were upregulated by *T. gondii* infection but were impaired by CCR5-deficiency (Additional file 1: Table S1). The functions of these differentially expressed genes (DEGs) were assessed by gene ontology (GO) term enrichment analysis (Table 1, Additional file 2: Table S2). Among the genes in which upregulation during infection was impaired by CCR5-deficiency, overrepresented GO terms were associated with immune response and response to stress categories. Kyoto Encyclopedia of Genes and Genomes (KEGG) pathway enrichment analysis showed that immune-related pathways such as the TNF signaling pathway, NOD-like signaling pathway, and antigen processing and presentation pathways were activated via CCR5 during the *T. gondii* infections (Additional file 3: Table S3; Additional file 4: Figure S1).

**Table 1:** Top 5 GO terms overrepresented in DEGs in which upregulation during *T. gondii* infection was affected by CCR5-deficiency. To explore the functions of DEGs in which upregulation during infection was affected by CCR5-deficiency, GO term enrichment analysis was performed

Cell type	Accession no.	FDR	# DEGs	# reference genes	GO term
Astrocyte	GO:0006952	8.1E-24	49	1416	defense response
	GO:0002376	5.7E-22	58	2282	immune system process
	GO:0006955	2.5E-21	45	1297	immune response
	GO:0051707	1.7E-18	34	879	response to other organism
	GO:0043207	1.7E-18	34	881	response to external biotic stimulus
Microglia	GO:0001819	4.8E-06	15	401	positive regulation of cytokine production
	GO:0002237	6.2E-05	12	359	response to molecule of bacterial origin
	GO:0006954	6.4E-05	16	646	inflammatory response
	GO:0001816	6.4E-05	17	704	cytokine production
	GO:0001817	6.9E-05	16	632	regulation of cytokine production

**Fig. 1:**
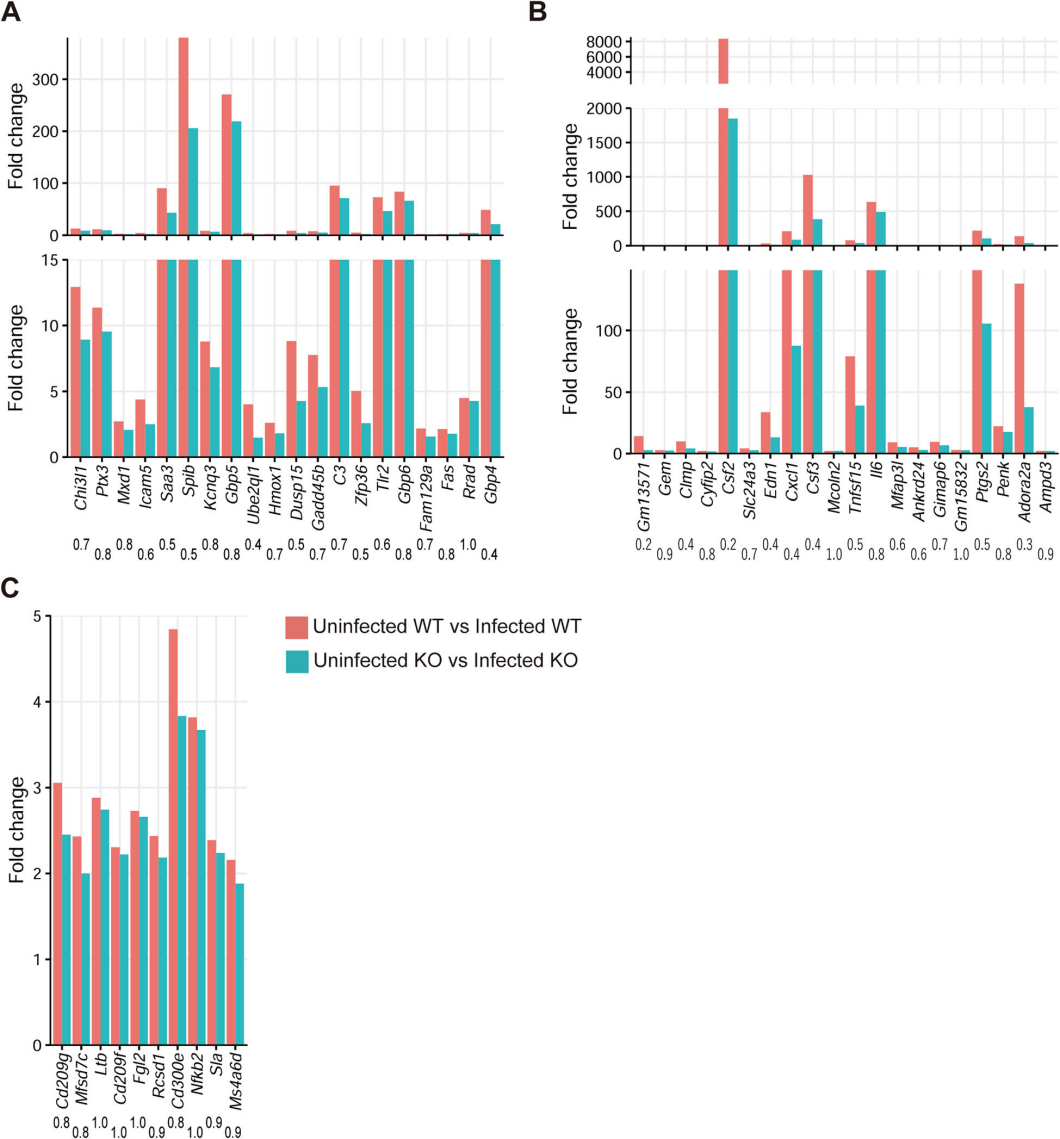
Expression of the top 20 DEGs in which upregulation during *T. gondii* infection was impaired by CCR5-deficiency. DEGs were ranked according to the fold-changes between the infected wild-type (WT) vs. infected CCR5-deficient (KO) mice. **a**, astrocytes; **b**, microglia; **c**, neurons. Each bar represents the fold-change between uninfected vs. infected mice. The values under the gene symbols are the ratio of the fold-change between uninfected KO vs. infected KO to that between uninfected WT vs. infected WT. The area of low fold-change is enlarged in panel A and B

To investigate the functions affected by CCR5-deficiency in astrocytes in more detail, DEGs in which upregulation during infection was impaired by CCR5-deficiency were ranked according to the fold-changes, calculated by DESeq2, occurring between *T. gondii-*infected cells from WT and CCR5KO mice, and the expression profiles of the top 20 genes were compared (Fig. 1a). The top 20 upregulated genes included IFN-γ inducible genes (i.e., *C3*, *Fas*, *Gbp4*, *Gbp5, Gbp6*). NF-κB target genes (i.e., *C3*, *Chi3l1*, *Fas*, *Gadd45β*, *Hmox1*, *Ptx3*, *Saa3*, *Tlr2*) were also abundant in DEGs in which upregulation during infection was impaired by CCR5-deficiency.

### Gene expression in microglia during *T. gondii* infection was affected by CCR5-deficiency

Primary microglia from WT and CCR5KO mice were infected with tachyzoites and subjected to RNA-seq followed by differential expression analysis. The analysis identified 93 genes that were upregulated by *T. gondii* infection but were impaired by CCR5-deficiency (Additional file 1: Table S1). The functions of these DEGs were reviewed by GO term enrichment analysis (Table 1, Additional file 2: Table S2). Among the genes in which upregulation during infection was impaired by CCR5-deficiency, overrepresented GO terms were associated with inflammatory response and cytokine production. In KEGG pathway enrichment analysis of these genes, immune-related pathways such as IL-17, TNF, and NF-κB signaling pathways were overrepresented (Additional file 3: Table S3, Additional file 5: Figure S2).

To better understand the functions affected by CCR5-deficiency in microglia, DEGs of upregulated genes during infection which were disrupted by CCR5-deficiency were ranked in the same way as described above, and the expression levels of the top 20 genes were compared (Fig. 1b). The top 20 upregulated DEGs included many NF-κB target genes (i.e., *Adora2a*, *Csf2*, *Csf3*, *Cxcl1 Edn1*, *Il6*, *Penk*, *Ptgs2*, *Tnfsf15*). In addition, CCR5-deficiency during infection affected the upregulation of cytokine genes such as *Csf2*, *Csf3*, *Cxcl1*, *Edn1*, and *Il6* and other genes associated with immune response (i.e., *Gimap6*, *Mcoln2*) and migration (i.e., *Cyfip2*).

### Gene expression in neurons during *T. gondii* infection was affected by CCR5-deficiency

Primary neurons from WT and CCR5KO mice were infected with tachyzoites and subjected to RNA-seq followed by differential expression analysis. The analysis identified only 10 genes in which upregulation was impaired by CCR5-deficiency and *T. gondii* infection (Additional file 1: Table S1). GO term enrichment and KEGG pathway analyses were not performed on neurons because the number of the DEGs was too small to estimate accurately.

To better understand the functions affected by CCR5-deficiency, the 10 DEGs of upregulated genes which were impaired by CCR5-deficiency were ranked in the same way as described above (Fig. 1c). These DEGs included genes related to the immune system or immune pathways (i.e., *Cd209g*, *Cd209f*, *Cd300e*, *Fgl2, Nfkb2*), signal transduction (i.e., *Cd300e*, *Ms4a6d*, *Nfkb2*, *Sla*), apoptosis (i.e., *Cd300e*, *Nfkb2*), T-cell activation (i.e., *Cd300e*, *Sla*), transcription factors (i.e., *Nfkb2*) and NF-κB targeted genes (i.e., *Cd209g*, *Nfkb2*).

### In vivo analysis of brain tissue from infected mice

**Fig. 2:**
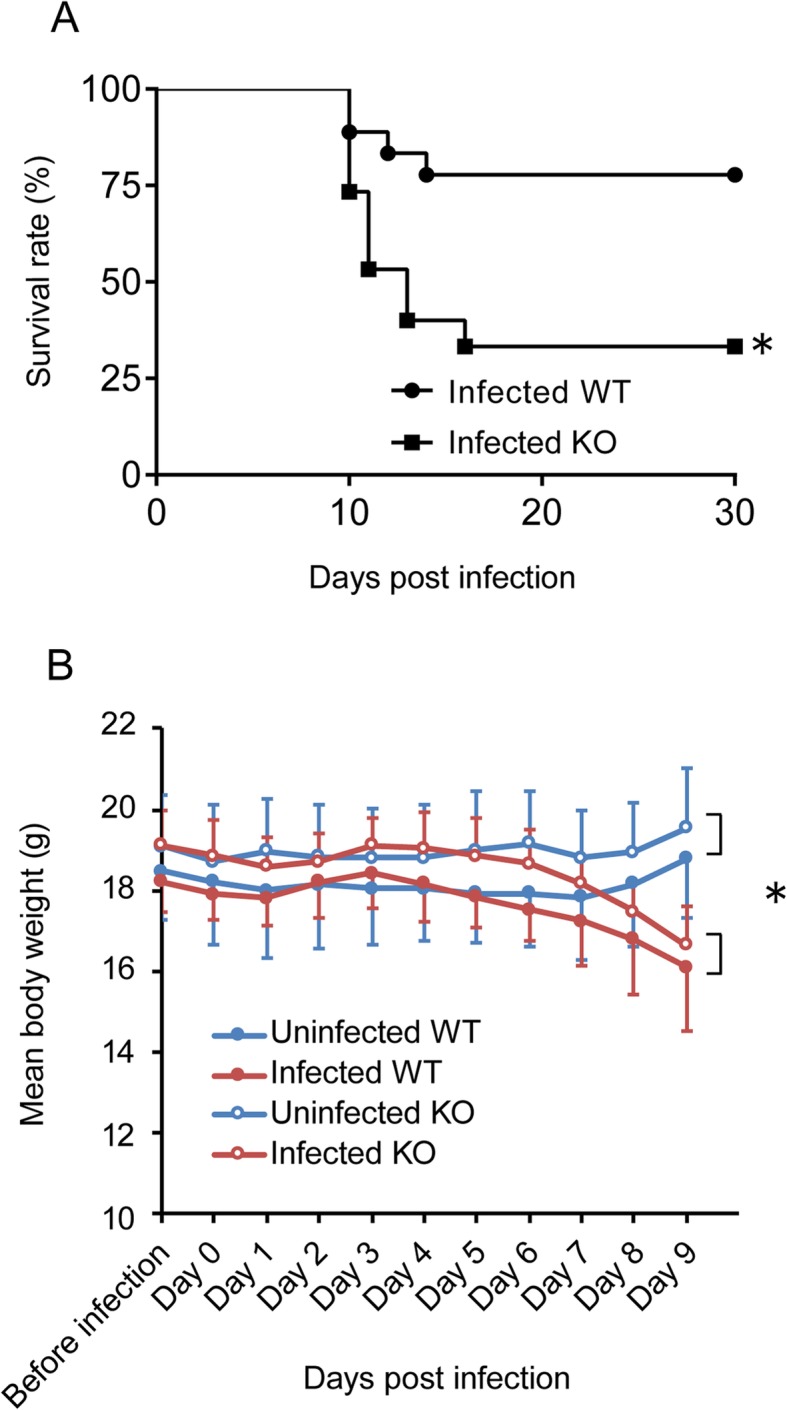
Survival rate and body weight of the infected wild-type (WT) and CCR5-deficient (KO) mice. **a** Survival rate was monitored until 30 dpi (infected WT, *n* = 10 + 8; infected CCR5KO, *n* = 9 + 6). Survival rate was compared between groups using the log-rank test (*p < 0.05). **b** Body weight change was monitored until 9 dpi, the day before the infected group started to die (uninfected WT, *n* = 4; uninfected CCR5KO, *n* = 4; infected WT, n = 10; infected CCR5KO, *n* = 9). Each point represents the mean ± SD. The weight difference between 0 dpi and 9 dpi was compared using two-way ANOVA. Effect of infection on body weight was significant (*p < 0.0001) but not the mouse genotype or the interaction between infection and mouse genotype (*p* > 0.05)

**Fig. 3:**
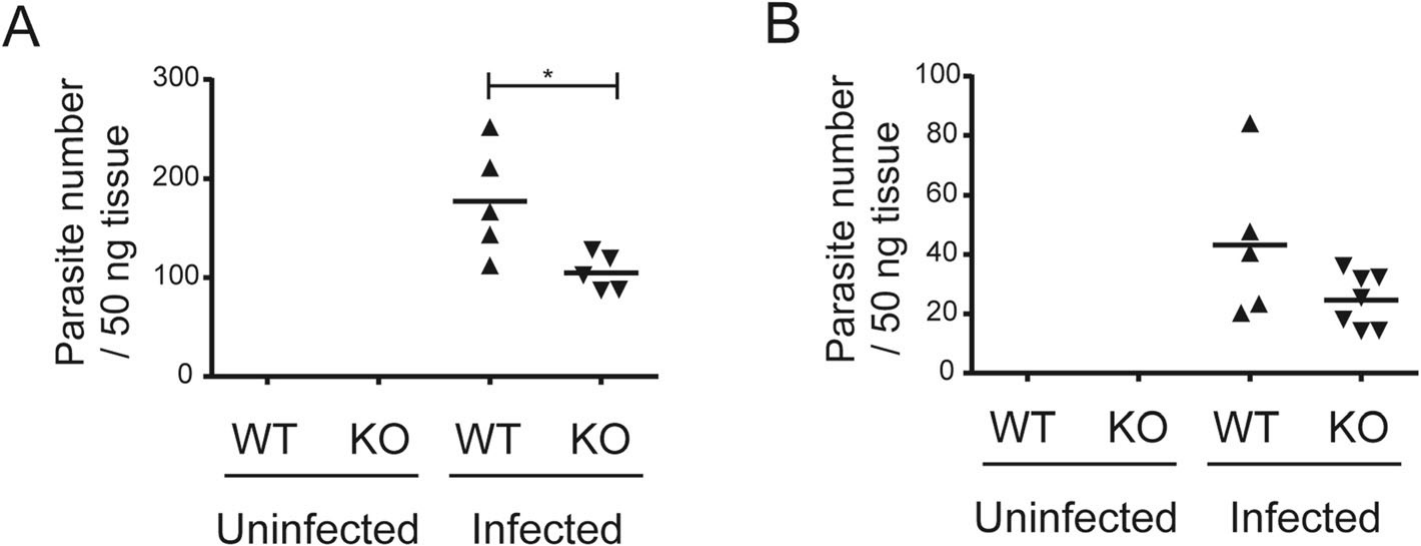
Parasite numbers in the brain in the in vivo experiment. Parasite numbers in the brain tissue from the *T. gondii*-infected wild-type (WT) and CCR5-deficient (KO) mice were determined by real-time quantitative PCR on the *T. gondii* B1 gene. **a**, 30 dpi; **b**, 7 dpi. Parasite numbers were compared using the student’s unpaired t-test between the WT and CCR5KO mice (*p > 0.05). C_t_ values higher than those for the uninfected samples were considered indicative of nonspecific amplification and were excluded from the plot. Each symbol represents the data point for one mouse, and the bars represent the mean value for all the group data points (uninfected WT, *n* = 3; uninfected CCR5KO, *n* = 3; infected WT, *n* = 5; infected CCR5KO, *n* = 7)

WT and CCR5KO mice were intraperitoneally injected with 1000 tachyzoites and monitored daily for 30 days. Both WT and CCR5KO mice died from 10 to 16 days post-infection (dpi), which is thought to be the acute phase of systemic infection with *T. gondii*. The survival rate at 30 dpi was significantly lower in the infected CCR5KO mice (3/9) than in the WT mice (8/10) (Fig. 2a). While the mouse body weight at 0 dpi did not differ significantly among four groups (i.e., uninfected WT, infected WT, uninfected CCR5KO, and infected CCR5KO), at 9 dpi, infection was found to have a significant effect on the body weight, but not the mouse genotype or the interaction between infection and mouse genotype (Fig. 2b). The number of parasites in the brain at 30 dpi was significantly lower in the CCR5KO mice than in the WT mice (*p* < 0.05) (Fig. 3a). Mice were also injected with a higher number of tachyzoites (5000 per mouse) and were sacrificed at 7 dpi. At the time point, there was no significant difference in the number of brain-located parasites between WT and CCR5KO (Fig. 3b).

**Fig. 4:**
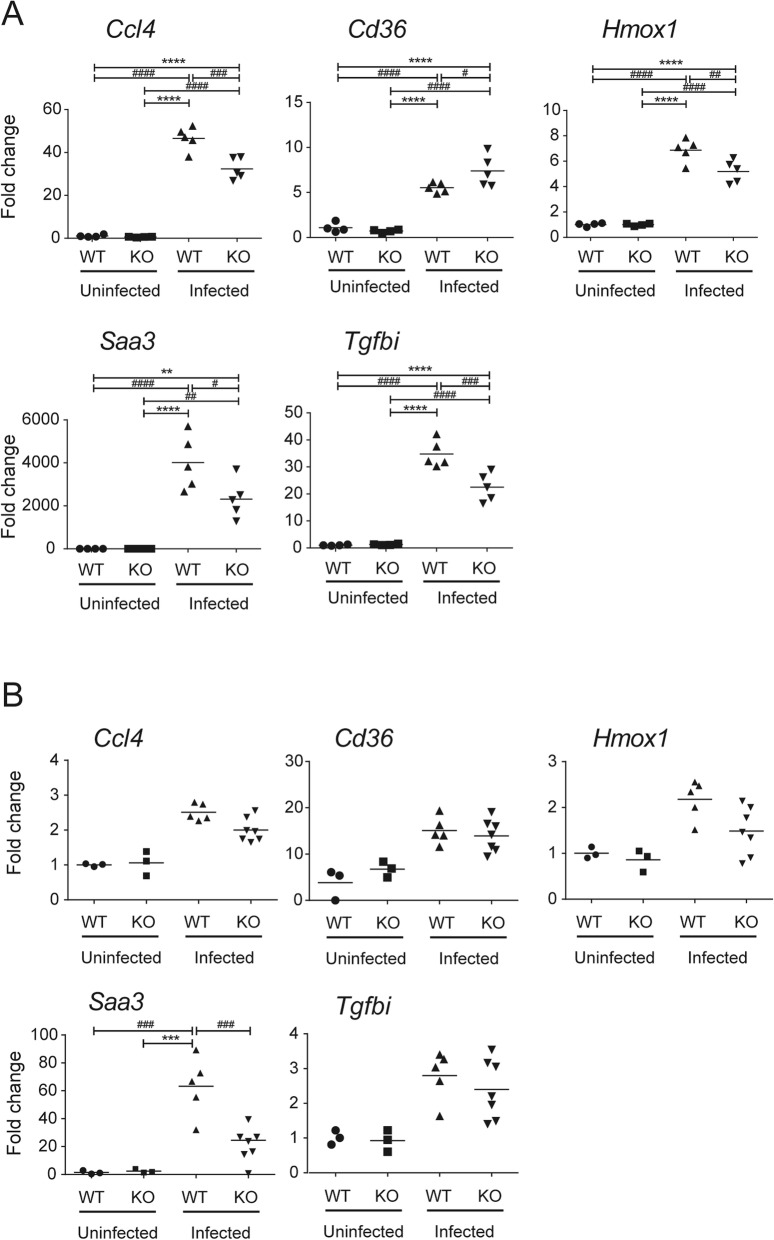
Expression of DEGs identified from the transcriptomic analysis was examined in the brain in the in vivo experiment. The brains of the *T. gondii*-infected wild-type (WT) and CCR5-deficient (KO) mice were prepared to determine their mRNA levels. **a**, 30 dpi; **b**, 7 dpi. The mRNA levels were normalized by the β-actin (*Actb*) mRNA level in the corresponding sample. * and # represent significant differences between the two groups in Tukey’s test after two-way ANOVA (**** and #### *p* < 0.0001, *** and ### *p* < 0.001, ** and ## *p* < 0.01, * and # *p* < 0.05). Each symbol represents the data point for one mouse, and the bars represent the mean value for all the group data points (uninfected WT, *n* = 4; uninfected CCR5KO, *n* = 4; infected WT, *n* = 5; infected CCR5KO, *n* = 5)

To investigate the in vivo expression of genes in which upregulation during infection was significantly impaired by CCR5-deficiency in the in vitro transcriptomic analysis, brain tissues from *T. gondii*-infected WT and CCR5KO mice were subjected to reverse transcription quantitative polymerase chain reaction (RT-qPCR) (Fig. 4, Additional file 6: Figure S3). Based on the results of the present transcriptomic profiling, 10 genes which were highly induced by *T. gondii* infection and showed a large difference between the fold-changes of “uninfected WT vs. infected WT” and “uninfected KO vs. infected KO” were selected for astrocytes (*Dusp15*, *Gbp4*, *Saa3*, *Spib*, *Ube2ql1*) and microglia (*Adora2a*, *Csf2*, *Csf3*, *Cxcl1*, *Ptgs2*). Moreover, we tested seven genes (*Chi3l1*, *Gbp5*, *Hmox1*, *Mcoln2*, *Nfkb2*, *Ptx3*, *Sla*) considering their functions in immune and defense responses. Furthermore, we selected another seven genes (i.e., *C1qa*, *Cd36*, *Gfap*, *Ifng*, *Irf4*, *Socs1*, *Tgfbi*), which we identified in our previous study to be associated with brain pathology during subacute *T. gondii* infection [20]. The genes encoding CCR5 (*Ccr5*) and its ligands (*Ccl3*, *Ccl4*, *Ccl5*) were also tested.

At 30 dpi, the expression of 5 genes (*Ccl4*, *Cd36*, *Hmox1*, *Saa3*, *Tgfbi*) showed a significant interaction between mouse genotype and *T. gondii* infection (*p* < 0.05), thus revealing the existence of a partly CCR5-dependent immune response in the *T. gondii*-infected brain (Fig. 4a). Among these five genes, the results for *Hmox1* and *Saa3* were consistent with the transcriptome analysis for astrocytes. In contrast, the gene expression pattern in the brain at 7 dpi was not in agreement with the transcriptome analysis results for each cell type, except for *Saa3* in astrocytes (Fig. 4b).

## Discussion

In this study, we profiled the gene expression patterns of primary brain cells exposed to *T. gondii* tachyzoites using RNA-seq. Genes in which upregulation during infection was impaired by CCR5-deficiency were subsequently examined in vivo to quantify their expression levels and to investigate their potential involvement in the control of parasite proliferation and pathology in the mouse brain.

For astrocytes, among the genes in which upregulation during infection was impaired by CCR5-deficiency, overrepresented GO terms and KEGG pathways were associated with immune response and response to stress categories. This suggested that CCR5 has a role in the immune response in astrocytes during *T. gondii* infection. The top 20 upregulated genes included IFN-γ inducible genes (i.e., *C3, Fas*, *Gbp4*, *Gbp5, Gbp6*). Guanylate binding protein (GBP) genes located on chromosome 3, including *Gbp5*, play a critical role in the destruction of the parasitophorous vacuole membrane (PVM), along with interferon-inducible immunity-related GTPases (IRGs) [5, 28]. *Gbp5* is IFN-γ inducible in astrocytes and participates in activating the nucleotide-binding oligomerization domain-like receptor protein 3 (NLRP3) inflammasome, a multiprotein oligomer responsible for activating inflammatory processes [29, 30]. *Gbp* genes located on chromosome 5, such as *Gbp4* and *Gbp6*, are also IFN-γ inducible, but their role in astrocytes and during *T. gondii* infection is not well studied. During viral infection, GBP4 negatively regulates virus-induced type I IFN and antiviral response by interacting with IFN regulatory factor (IRF) 7 [31]. CCR5 is probably associated with parasite elimination by these GBPs in astrocytes. It is still unclear whether these IFN-γ inducible genes were induced with or without IFNs. Among the IFNs, only IFN-β (encoded by *Ifnb1*) was detected in RNA-seq in astrocytes although the mean normalized counts were quite low (4.5 ± 2.0 in infected WT and 1.8 ± 3.0 in infected KO). IFN-β is known to induce some anti-*T. gondii* genes [32]. Another possibility is that the IFN-γ inducible genes were induced without IFNs. It is known that some IFN-γ inducible genes are induced by signal transducers and activators of transcription 1 (STAT1), and *T. gondii* can phosphorylate STAT1 without IFNs [33]. Our results suggest that the mechanisms used for killing intracellular parasites partially depend on CCR5 in astrocytes.

In addition, NF-κB target genes (i.e., *C3*, *Chi3l1*, *Fas*, *Gadd45β*, *Hmox1*, *Ptx3*, *Saa3*, *Tlr2*) were abundant in the DEGs in which upregulation during infection was impaired by CCR5-deficiency. The current results suggest that signal transduction via CCR5 leads to activation of NF-κB during *T. gondii* infection in astrocytes, although CCR5 involvement in NF-κB activation is complicated as follows. The HIV envelope gp120 protein reportedly activates NF-κB via CCR5, but CCL4, a natural ligand for CCR5, did not activate NF-κB [34]. Another ligand, CCL5, activates NF-κB and induces cell migration in human osteosarcoma [35].

For microglia, among the DEGs which were upregulated during infection and were impaired by CCR5-deficiency, overrepresented GO terms were associated with immune response and cytokine production, and KEGG pathway enrichment analysis showed immune-related pathways such as IL-17, TNF, and NF-κB signaling pathways. CCR5 may participate in some of the functions in microglia such as cell processes including the regulatory processes essential for tissue development, architectural refinement, neural environment maintenance, response to injury and subsequent remodeling and repair [36]. A previous study showed that CCR1, CCR5, and CXCR2 chemokine receptors were functionally expressed on microglia [37]. With activated microglia, CCR5 is suggested to be responsible for changing the secretion levels of IL-10, insulin-like growth factors, and nitric oxide (NO) when engaged by CCL5 [37]. Our GO term analysis results regarding DEGs in which upregulation during infection was impaired by CCR5-deficiency are similar to previous reports about microglia functions [36, 37], suggesting that CCR5 has an effect on immune responses in microglia during *T. gondii* infection.

The top 20 upregulated DEGs in microglia included many NF-κB target genes (i.e., *Adora2a*, *Csf2*, *Csf3*, *Cxcl1 Edn1*, *Il6*, *Penk*, *Ptgs2*, *Tnfsf15*). Among the NF-κB target genes, *Csf2*, *Csf3*, *Cxcl1*, *Edn1*, and *Il6* are cytokines. *Cxcl1*, a pro-inflammatory cytokine, is a known M1 macrophage-producing chemokine [38]. In M1 cells, the *Toxoplasma* dense granule protein GRA15-dependent induction of *Cxcl1* has been reported [39]. Microglia and macrophages are mesodermal in origin [40], and pro-inflammatory M1 microglia may be capable of expressing this chemokine. Granulocyte-macrophage colony-stimulating factor (encoded by *Csf2*) has numerous effects on microglia, ranging from induction of proliferation to changes in morphology, and also increases lipopolysaccharide-induced production of proinflammatory mediators via TLR4 [41, 42]. Granulocyte colony-stimulating factor (encoded by *Csf3*), which was also identified as a factor produced by Toll-like receptor (TLR) 4-activated microglia, might contribute to oligodendrocyte progenitor cell proliferation or differentiation [43]. Because *T. gondii* is recognized by host TLRs including TLR4 [44], CCR5 may be involved in a pathogen recognition mechanism in microglia via TLR4. Thus far, the downstream signaling pathway via CCR5 remains obscure, especially in brain cells. Our results suggest that CCR5 may be involved in NF-κB activation in microglia during *T. gondii* infections, leading to expression of pro-inflammatory cytokines and chemokines. Furthermore, a previous study showed that deletion of NF-κB signaling in microglia rescued motor neurons from microglial-mediated death in vitro and extended survival in mice with amyotrophic lateral sclerosis by impairing pro-inflammatory microglial activation [45]. It is possible that activating NF-κB in microglia leads to tissue injury in the *T. gondii*-infected brain and causes neurological dysfunctionality. Future studies on the signaling pathway downstream of CCR5 and its relationship with NF-κB will provide better insight into the detailed mechanism underlying the functional role of CCR5.

Upregulation of some immune-related genes in neurons was also impaired by CCR5-deficiency. Among the upregulated DEGs in neurons, *Nfkb2* and *Cd209g* are NF-κB target genes. *Nfkb2*, which encodes the NF-κB p100 subunit, is required for the canonical activation pathway. A direct role for *Nfkb2* in the immune response against *T. gondii* has been described in the *Nfkb2*^−/−^ mouse model, in which severe *Toxoplasma* encephalitis develops [46]. Murine CD209g shares structural and sequence homology with human CD209, which belongs to the family of C-type lectins that participate in innate immune responses by binding and clearing pathogens. In neurons, however, the level of perturbation by CCR5-deficiency was quite low, and the number of genes upregulated in a partly CCR5-dependent manner was much smaller compared with the results in astrocytes and microglia. Although this result might be because of the different multiplicity of infection, there is another possibility that involvement of CCR5 in the immune response to *T. gondii* infection in neurons differs from that in astrocytes and microglia, and that the host defense mechanism in neurons is less dependent on CCR5 than in astrocytes and microglia during infection. NF-κB has diverse functions, depending on the cellular context. Constitutively activated NF-κB is detected mostly in neurons, whereas NF-κB in glia has a lower basal activity and is heavily inducible. NF-κB function in neurons is responsible for learning and memory rather than inflammatory processes [47]. This could have something to do with neurons as a primary target cell of chronic infection of *T. gondii*. The roles performed by the upregulated DEGs against *T. gondii* infection in neurons warrant further study.

In in vivo assays, our result showed no significant difference in the number of brain-located parasites at 7 dpi between the WT and CCR5KO mouse groups. This result at 7 dpi is consistent with a previous study [24]. On the other hand, at 30 dpi, the number of parasites in the brain was significantly lower in the CCR5KO mice than in the WT mice. Contrary to our result, a previous report revealed that cyst numbers in the brain were higher in CCR5KO mice (C57BL/6 × 129 background) than in WT mice [22]. In addition to the difference in the background of mice, the difference between the present and previous studies could be due to the different methodology: timing of brain collection (30 dpi vs. at 18 dpi); parasite strain used (PLK strain vs. ME-49 strain); parasite life stage (1000 tachyzoites vs. 20 cysts). The susceptible mice died during the acute phase of *T. gondii* infections, which may be why the CCR5KO-infected mice harbored fewer parasites than the WT mice. Moreover, the CCR5KO mice showed significantly lower survival rate after infection. This may be due to the impaired immune response outside the brain; thus, we did not focus on the different survival rates in this study. Furthermore, our results revealed no significant differences in the number of brain-located parasites at 7 dpi between WT and CCR5KO.

Our current transcriptome analysis for primary brain cells showed that some genes were considered to be at least partly involved in the CCR5-dependent cellular response, which simultaneously suggests the involvement of CCR5 in brain pathology during *T. gondii* infection. *Saa3*, which was induced during acute and chronic inflammatory responses, encodes one of the primary acute-phase reactants, serum amyloid A (SAA) proteins. SAA is reported to prime NLRP3-inflammasome-dependent responses in macrophages [48, 49] and synovial fibroblasts [50], but its effect on brain glia is unknown [51]. *Saa3* is predominantly expressed in macrophages [52], and the mRNA is induced in many tissues and is expressed at various levels in several different cell types [53]. Macrophages are a principal cell type that express extrahepatic *Saa3* [53]. In the present study, *Saa3* was upregulated in the brain tissues of *T. gondii*-infected mice, which might be related to its expression level in astrocytes and/or infiltrated peripheral blood immune cells. At 7 dpi, *Saa3* expression in the brain was probably upregulated in response to the immune response against systemic infection with *T. gondii*, and this response possibly continued until 30 dpi. From the acute to subacute phase, *Saa3* may play a role in promoting inflammation, similar to that which occurs in cancer metastasis [54], which is likely to depend on CCR5.

Upregulation of *Hmox1* during infection with *T. gondii* was also impaired by CCR5-deficiency at 30 dpi. The protein encoded by the *Hmox1* gene participates in cellular defenses against oxidative stress [55]. In response to an immune stimulus, glial cells, specifically astrocytes and microglia, become activated in the process termed gliosis [56] and stimulate the acute-phase response characterized by the release of cytokines, proteases, prostaglandins, and NO via increased expression of iNOS, and by stimulating the arachidonic acid cascade with increased production of reactive oxygen species (ROS) [57]. Oxidative stress injury is consistently linked to bipolar disease and toxoplasmosis. A cross-sectional study has investigated the possible role of *Toxoplasma*-induced oxidative stress in the development of bipolar disorders [58]. Also, *Hmox1* is reportedly associated with cellular protection against oxidative stress [59] and indoleamine 2,3-dioxygenase (IDO) induction [60]. These findings suggest that *Hmox1* may help to protect host cells from oxidative stress via an anti-parasitic mechanism (e.g., NO production and ROS production) [61] and that the attenuation may be partly dependent on CCR5. Meanwhile, most of the tested genes showed a low consistency between in vitro and in vivo experiments. This could be due to the different length of post-infection time between experiments (24 h vs. ≥7 days). We set the time points because our preliminary data showed that it takes about 7 days for parasite tachyzoites to start the invasion into the brain after intraperitoneal injection, but it seems there are still some gaps between in vitro and in vivo. Further studies would be required to clarify these findings.

## Conclusions

This study is the first transcriptomic report on the relationship between CCR5 and *T. gondii* infection in primary brain cells and brain tissue. During *T. gondii* infection, expression levels of many genes associated with the immune response were affected by CCR5-deficiency. Our transcriptome result from purified primary cell cultures has provided new insight into the onset of local infection in each brain cell type and the involvement of CCR5. Many other factors could also influence the effect of *T. gondii* infection in brain tissue, including infiltration of immune cells from peripheral blood and cell-cell communications. We did not perform perfusion to remove the components in the peripheral blood due to a technical limitation, which might have resulted in an underestimation of changes in the brain in this study.

Because of its biological and anatomical complexity, in this study, the brain tissue showed expression patterns different from those of the primary brain cells, and differential expression by CCR5-deficiency was hardly detectable during the acute infection phase (7 dpi). In a tissue-level experiment, the effect of CCR5-deficiency on a cellular level should be covered and compensated, which might have contributed to our difficulty in observing gene expression difference between WT and CCR5KO mice in vivo. Nevertheless, our overall results suggest the involvement of CCR5 in the mouse brain during the subacute infection phase (30 dpi). Notably, the expression patterns of *Saa3* and *Hmox1* were consistent with the transcriptome analysis and the in vivo study at 30 dpi. This is the first study documenting the participation of these two genes in the CCR5 signaling pathway. Our data, which provides essential information on the involvement of CCR5 in neurological alteration and host protection from *T. gondii*, will assist further studies in elucidating its precise function.

## Methods

### Animals

C57BL/6 J mice were obtained from Clea Japan (Tokyo, Japan) and used as WT mice in this study. CCR5KO mice (B6.129P2-Ccr5^tmlKuz^/J, Stock No. 005427) were purchased from the Jackson Laboratory (Bar Harbor, ME, USA). Differential gene expressions due to the different parental strains were minimized by backcrossing the stock of CCR5KO mice to C57BL/6 eleven times in total before being imported into our laboratory. For the initial preparations of brain cells, mice at 6–8 weeks old were used as parents. Mice of 9–10 weeks old were used for in vivo experiments. All animals were housed in cages (6 mice/cage, 225 mm × 340 mm × 155 mm) with wood chips under specific-pathogen-free conditions in the animal facility of the National Research Center for Protozoan Diseases at Obihiro University of Agriculture and Veterinary Medicine, Hokkaido, Japan.

### Preparation of *T. gondii* tachyzoites

*T. gondii* tachyzoites (PLK strain, type II) were maintained by serial passage on Vero cell monolayers at 37 °C in humidified air with 5% CO_2_. Parasites were washed by centrifugation, host cell debris was removed, and the parasites were resuspended in cold phosphate-buffered saline. Clustered cells and debris were removed by repeatedly passing the suspension through a 27-gauge needle and filtration through a 5.0-μm pore-size filter (Millipore, Burlington, MA, USA).

### Cultures of astrocytes, microglia and neurons

Astrocytes, microglia and neurons were obtained from the brain cortices of fetal mice (age, E17–18) according to a previously described procedure [62, 63], with some modifications. Further details of the cultures can be found in Additional file 7: Supplemental Methods.

### In vitro infection and RNA extraction

Primary brain cells were infected with *T. gondii* tachyzoites suspended in the medium appropriate for each cell type. Multiplicities of infection were 0.2, 1.0, and 1.0 for neurons, astrocytes, and microglia, respectively. Infected and uninfected groups of cells were prepared in triplicate wells for each mouse genotype and each cell type. After 24 h of infection, total RNA was extracted from individual wells of the monolayer cell samples with TRI reagent (Sigma-Aldrich, St. Louis, MO, USA), according to the manufacturer’s protocol. RNA extracted from each well was individually subjected to RNA-seq.

### RNA-seq analysis

Transcriptome sequencing was performed according to the protocol used in our previous study [20]. Briefly, 1 μg of total RNA was subjected to poly-A selection. Sequencing libraries were constructed using the TruSeq RNA Sample Prep Kit (Illumina, San Diego, CA, USA), while 36-bp single-end sequencing was performed using the Illumina Genome Analyzer IIx and TruSeq SBS Kit v5-GA (36-cycle) (Illumina), according to the manufacturer’s instructions. All treatments and subsequent analyses were performed on individual transcripts. The data of uninfected samples has also been used in our previously published article [64] because all the sample cells in this article were prepared in the same experimental batch in the previous article.

### Identification of DEGs in which upregulation during *T. gondii* infection was impaired by CCR5-deficiency

Sequence tags were aligned using TopHat (version 1.3.3 doi:10.1093/bioinformatics/btp120) and gene transfer format (gtf) data (Mus_musculus.GRCm38.69), as previously described [20]. Raw sequence reads were mapped to the mouse genome (mm10, http://hgdownload.cse.ucsc.edu/downloads.html#mouse) with a two mismatches allowance. Based on the mapping results, differential expression analysis was performed for pairwise comparisons of the expression data using the R packages DESeq2 [65] and edgeR [66, 67]. In this study, only genes which appeared differentially expressed in both DESeq2 and edgeR were considered as DEGs. DEGs in which upregulation during infection was impaired by CCR5-deficiency were defined as follows: (1) log2 fold-change > 1 and FDR < 0.05 between the infected and uninfected WT mice; (2) FDR < 0.05 between the infected WT and CCR5KO mice; (3) the fold-change was higher in “uninfected WT vs. infected WT” than in “uninfected KO vs. infected KO”. Minor expressed genes were excluded by using cut-off values of ≤100 mean normalized counts in the infected WT mice for upregulated genes. As we aimed to investigate the effect of CCR5 on the expression of genes during *T. gondii* infection, we focused on DEGs in which upregulation during *T. gondii* infection was impaired by CCR5-deficiency although some genes were up/downregulated just by CCR5-deficiency regardless of *T. gondii* infection (Additional file 8: Table S4). For comparison among four groups (i.e., uninfected WT, infected WT, uninfected CCR5KO, and infected CCR5KO), the raw-read counts for each gene were normalized by the iDEGES method implemented in the TCC package [68]. Mouse Genome Informatics (MGI) data were obtained from the Mouse Genome Informatics database [69]. The expression of *Ccr5* in each cell type was shown in Additional file 9: Figure S4.

### GO analysis of DEGs in which upregulation during *T. gondii* infection was impaired by CCR5-deficiency

The DEGs in which upregulation during *T. gondii* infection was impaired by CCR5-deficiency were functionally categorized by GO term enrichment analysis. Statistical overrepresentation of GO terms for selected genes was compared with the reference genes (all genes; 37,315 genes) using the goseq package in R software [70]. Genome-wide annotation for mouse was obtained using the org.Mm.eg.db package [71]. Functional annotation charts for the enriched GO terms were generated using the GO terms associated with biological processes. Only GO terms with FDRs < 0.05 and the number of DEGs ≥10 were considered to represent functional enrichment.

### KEGG pathway enrichment analysis

The KEGG database is a bioinformatics tool which assembles large-scale molecular datasets, like gene lists, into biological pathway maps [72]. The host DEGs list was subjected to KEGG pathway enrichment analysis using the clusterProfiler package [73] in the statistical environment R to assess their overarching functions. After counts per million normalization, the expression level of each gene in the enriched pathway was normalized by Z-score normalization and then visualized. Normalized gene expression was visualized in a heatmap using the heatmap.2 function [74] in the gplots package in R. Genes were hierarchically clustered based on the 1-Pearson correlation distance and group average method.

### In vivo infections and brain collections

WT and CCR5KO mice were anesthetized with isoflurane (4% for 1 min) and then intraperitoneally injected with 1000 tachyzoites suspended in 400 μL of Roswell Park Memorial Institute (RPMI)-1640 Medium (Sigma-Aldrich). Their survival was monitored until 30 dpi. Each strain of mice was randomly allocated to uninfected and infected groups. Mice in uninfected groups were injected with RPMI-1640 Medium only. Data from two independent experiments were combined (uninfected WT, *n* = 4 + 4; uninfected CCR5KO, n = 4 + 4; infected WT, *n* = 8 + 10; infected CCR5KO, *n* = 6 + 9). In the second experiment, the mice body weights were monitored daily (uninfected WT, n = 4; uninfected CCR5KO, n = 4; infected WT, *n* = 10; infected CCR5KO, *n* = 9). After the experimental period, surviving mice were euthanized by cervical dislocation and then dissected. A half brain from each surviving mouse was harvested at 30 dpi (uninfected WT, n = 4; uninfected CCR5KO, n = 4; infected WT, *n* = 5; infected CCR5KO, n = 5), homogenized with BioMasher I (Nippi, Tokyo, Japan) and then subjected to RNA extraction with 2.5 ml of TRI reagent following the manufacturer’s protocol for tissue sample preparation and RNA isolation. The complementary DNA (cDNA) was prepared from 400 ng of RNA per reaction using PrimeScript™ II 1st strand cDNA Synthesis Kit (Takara Bio Inc., Shiga, Japan), following the manufacturer’s instructions.

Additionally, WT and CCR5KO mice were infected by intraperitoneal injections with a higher dose of tachyzoites (5000 tachyzoites per mouse) attempting to make the difference between WT and CCR5KO clear. The brains from these mice were harvested at 7 dpi (acute phase) when *T. gondii* tachyzoites typically start to invade into the brain. A half brain from each mouse was subjected to RNA extraction and cDNA preparation, as described above. The number of mice per group was as follows: uninfected WT, *n* = 3; uninfected CCR5KO, n = 3; infected WT, n = 9; infected CCR5KO, *n* = 8.

### Reverse transcription quantitative PCR (RT-qPCR) for brain tissue analysis

The relative mRNA levels were calculated as described previously [64]. Primer sequences used for RT-qPCR were listed in Additional file 10: Table S5. Further details of the analyses can be found in Additional file 7: Supplemental Methods.

### DNA extraction, quantitative PCR and *T. gondii* detection

The parasite number was calculated as described previously [64]. Further details of the analyses can be found in Additional file 7: Supplemental Methods.

### Statistical analysis

Survival rate was compared between the WT and CCR5KO groups using the log-rank test. Regarding body weight change, weight differences between 0 dpi and 9 dpi were analyzed using two-way analysis of variance (ANOVA). Parasite numbers in the brain tissue were compared between the WT and CCR5KO mice at each dpi using the student’s unpaired t-test. C_t_ values higher than those for the uninfected samples were considered indicative of nonspecific amplification and were excluded from the analysis. Fisher’s exact test was performed to compare the percentage of mice with detectable brain parasites. mRNA levels were normalized against the β-actin (*Actb*) mRNA level in the corresponding sample and were compared between the WT and CCR5KO groups using Tukey’s test after two-way ANOVA.

## Supplementary information

**Additional file 1: Table S1.** Detailed expression data for DEGs in which upregulation during *T. gondii* infection was impaired by CCR5-deficiency. (XLSX 67 kb)

**Additional file 2: Table S2.** Detailed data for top 10 GO terms overrepresented in the DEGs in which upregulation during *T. gondii* infection was impaired by CCR5-deficiency in astrocytes and microglia. (XLSX 12 kb)

**Additional file 3: Table S3.** Top10 KEGG pathways enriched in the DEGs in which upregulation during *T. gondii* infection was impaired by CCR5-deficiency in astrocytes and microglia. (XLSX 11 kb)

**Additional file 4: Figure S1.** Expression patterns of genes in the TNF signaling pathway in astrocytes. The enlarged area shows the genes thought to be affected by CCR5-deficiency. (TIF 826 kb)

**Additional file 5: Figure S2.** Expression patterns of genes in the IL-17 signaling pathway in microglia. The enlarged area shows the genes thought to be affected by CCR5-deficiency. (TIF 874 kb)

**Additional file 6: Figure S3.** Gene expression in the brain of *T. gondii*-infected mice. Genes with no significant interactions between mouse genotype and infection in a two-way ANOVA are shown. Each symbol represents the data point for one mouse, and the bars represent the mean value for all the group data points. A, 30 dpi (uninfected wild-type mice (WT), *n* = 4; uninfected CCR5-deficient mice (CCR5KO), n = 4; infected WT, *n* = 5; infected CCR5KO, *n* = 5); B, 7 dpi (uninfected WT, *n* = 3; uninfected CCR5KO, n = 3, infected WT, *n* = 5; infected CCR5KO, *n* = 7). (TIF 970 kb)

**Additional file 7:** Supplemental Methods. (DOCX 26 kb)

**Additional file 8: Table S4.** Genes up/downregulated just by CCR5-deficiency regardless of *T. gondii* infection. DEGs were identified by comparing between the uninfected wild-type and uninfected CCR5-deficient mice, with a threshold of 2-fold change and < 0.05 false discovery rate. (XLSX 114 kb)

**Additional file 9: Figure S4.** Expression of *Ccr5* in astrocytes, microglia, and neurons in the transcriptomic analysis. A, astrocytes; B, microglia; C, neurons. Each bar represents the mean ± SD (*n* = 3), which were calculated after normalizing the raw-read counts using the iDEGES method. (TIF 1167 kb)

**Additional file 10: Table S5.** Primer sequences used in RT-qPCR. (XLSX 11 kb)

**Additional file 11:** The ARRIVE Guidelines Checklist. (PDF 1093 kb)

## Data Availability

All data generated or analyzed during this study are included in this published article and its supplementary information files. The datasets are also available from the corresponding author on reasonable request.

## Abbreviations

AIDS: Acquired immune deficiency syndrome
ANOVA: Analysis of variance
CCL: C-C chemokine ligand
CCR: C-C chemokine receptor
cDNA: Complementary DNA
CNS: Central nervous system
CYFIP2: Cytoplasmic FMR1 interacting protein 2
DEG: Differentially expressed gene
Dpi: Days post infection
FDR: False discovery rate
GBP: Guanylate binding proteins
GO: Gene ontology
Gtf: Gene transfer format
IDO: Indoleamine 2,3-dioxygenase
IFN: Interferon
IL: Interleukin
iNOS: Inducible nitric oxide synthase
IRF: Interferon regulatory factor
IRG: Inducible immunity related GTPase
KEGG: Kyoto Encyclopedia of Genes and Genomes
KO: Knock-out
MGI: Mouse Genome Informatics
NF-κB: Nuclear factor-kappa B
NLRP3: Nucleotide-binding oligomerization domain-like receptor protein 3
NO: Nitric oxide
PVM: Parasitophorous vacuole membrane
RNA-seq: RNA sequencing
ROS: Reactive oxygen species
RPMI: Roswell Park Memorial Institute
RT-qPCR: Reverse transcription quantitative chain polymerase reaction
SAA: Serum amyloid A
STAT1: Signal transducers and activators of transcription 1
TLR: Toll-like receptor
WT: Wild-type
