# Supplementary figures and images for: Transcriptome analysis of the effect of C-C chemokine receptor 5 deficiency on cell response to Toxoplasma gondii in brain cells
Source: BMC Genomics. 2019 Sep 11;20:705. doi: 10.1186/s12864-019-6076-4 (PMC6737708; doi:10.1186/s12864-019-6076-4)

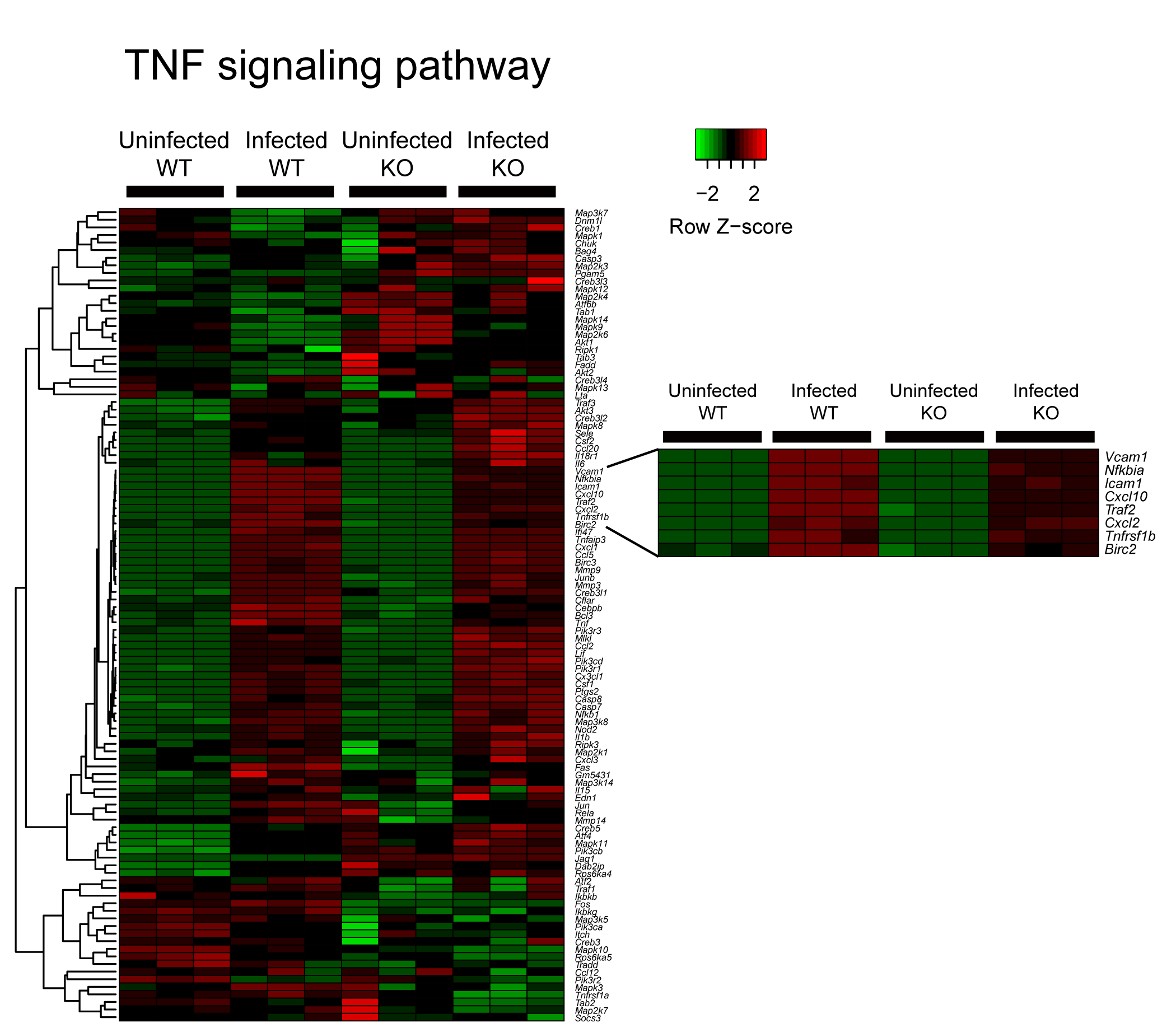

Supplement: Supplementary file 4 — Additional file 4: Figure S1. Expression patterns of genes in the TNF signaling pathway in astrocytes. The enlarged area shows the genes thought to be affected by CCR5-deficiency. (TIF 826 kb) [file 12864_2019_6076_MOESM4_ESM.tif]

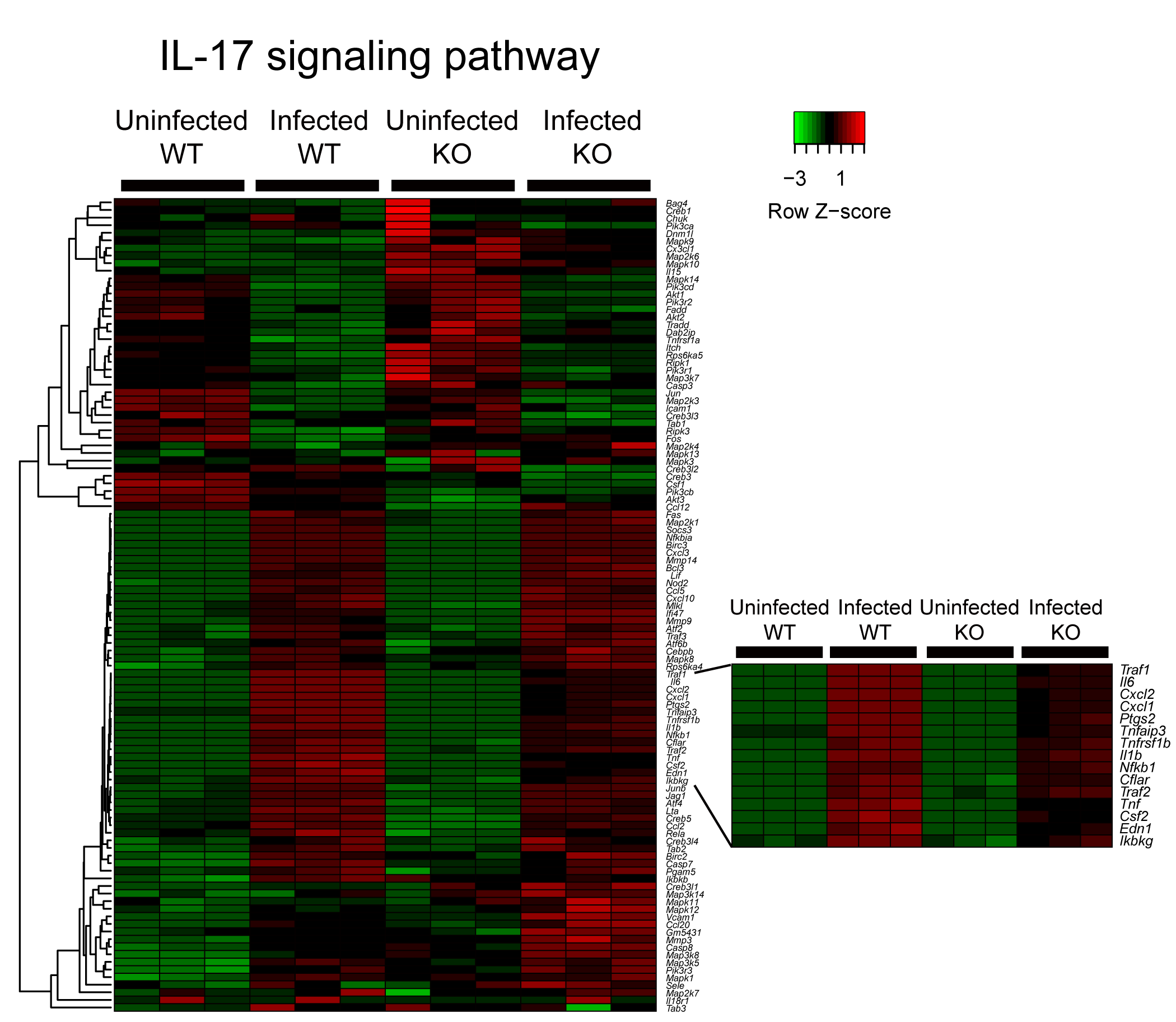

Supplement: Supplementary file 5 — Additional file 5: Figure S2. Expression patterns of genes in the IL-17 signaling pathway in microglia. The enlarged area shows the genes thought to be affected by CCR5-deficiency. (TIF 874 kb) [file 12864_2019_6076_MOESM5_ESM.tif]

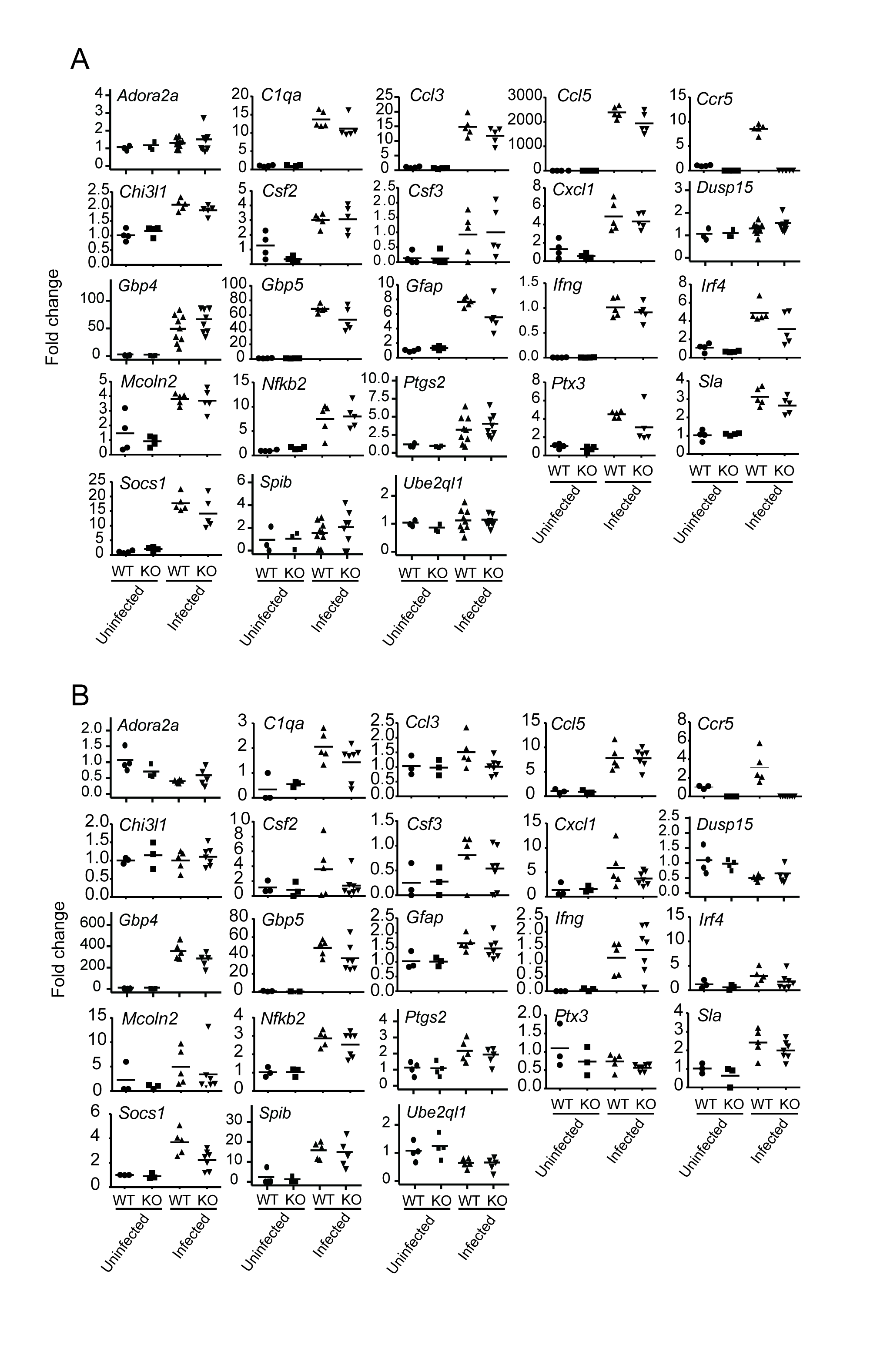

Supplement: Supplementary file 6 — Additional file 6: Figure S3. Gene expression in the brain of T. gondii-infected mice. Genes with no significant interactions between mouse genotype and infection in a two-way ANOVA are shown. Each symbol represents the data point for one mouse, and the bars represent the mean value for all the group data points. A, 30 dpi (uninfected wild-type mice (WT), n = 4; uninfected CCR5-deficient mice (CCR5KO), n = 4; infected WT, n = 5; infected CCR5KO, n = 5); B, 7 dpi (uninfected WT, n = 3; uninfected CCR5KO, n = 3, infected WT, n = 5; infected CCR5KO, n = 7). (TIF 970 kb) [file 12864_2019_6076_MOESM6_ESM.tif]

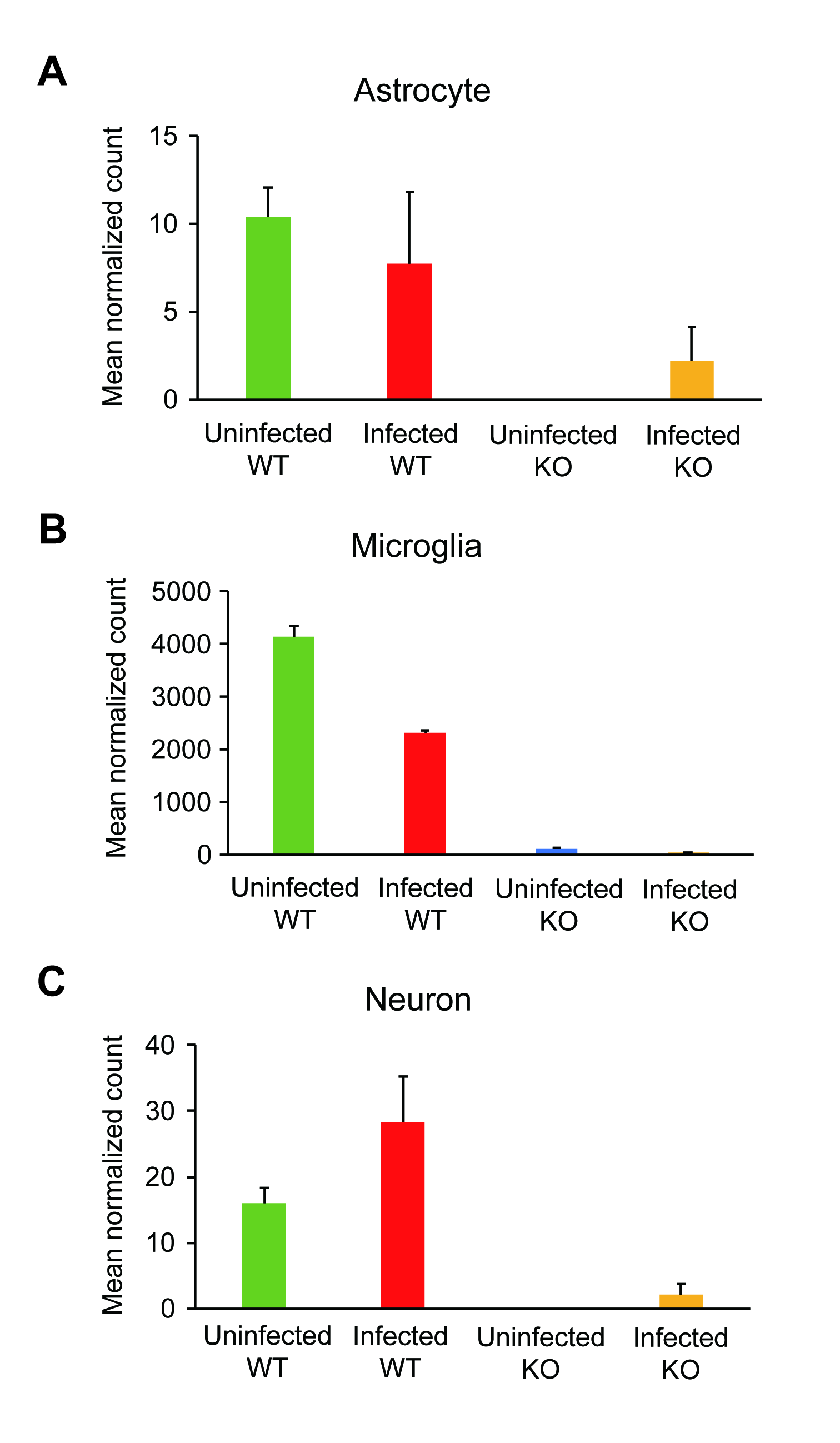

Supplement: Supplementary file 9 — Additional file 9: Figure S4. Expression of Ccr5 in astrocytes, microglia, and neurons in the transcriptomic analysis. A, astrocytes; B, microglia; C, neurons. Each bar represents the mean ± SD (n = 3), which were calculated after normalizing the raw-read counts using the iDEGES method. (TIF 1167 kb) [file 12864_2019_6076_MOESM9_ESM.tif]
