# Supplementary material for: Transcriptome analysis of the effect of C-C chemokine receptor 5 deficiency on cell response to Toxoplasma gondii in brain cells
Source: BMC Genomics. 2019 Sep 11;20:705. doi: 10.1186/s12864-019-6076-4 (PMC6737708; doi:10.1186/s12864-019-6076-4)
Supplement: Supplementary file 7 — Additional file 7: Supplemental Methods. (DOCX 26 kb) [file 12864_2019_6076_MOESM7_ESM.docx]

# Supplemental methods

## Astrocyte cultures

Astrocytes were obtained from the brain cortices of fetal mice (age, E17–18) according to a previously described procedure [1], with some modifications. The fetal mice were decapitated, and their brains were removed. After removing the meninges, the cortices were mechanically dissociated in Dulbecco's Modified Eagle’s Medium (DMEM; Sigma-Aldrich, St Louis, MO, USA) containing 0.25% trypsin and 0.01% DNase to obtain a single-cell suspension. After incubation at 37°C for 10 min, the dissociated cells were washed and suspended in DMEM/F-12 (Gibco-BRL, Grand Island, NY, USA) supplemented with penicillin-streptomycin (100 U/ml penicillin and 100 µg/mL streptomycin; Sigma-Aldrich), 10% fetal bovine serum (FBS; Columbia Biosciences, Columbia, MD, USA), and G-5 Supplement (Gibco-BRL). The cells were plated in 75-cm^2^ flasks (density, 2 × 10^6^ cells/flask) and then incubated at 37°C in humidified 5% CO2 (95% air atmosphere). The culture medium was changed every 3 days until the cultures reached confluence, usually after 7–8 days. Astrocyte monolayers were washed and dissociated with 0.25% trypsin–EDTA solution. The dissociated astrocytes were centrifuged (4°C, 500 × *g*, 5 min), washed in DMEM/F-12 supplemented with 10% FBS and G-5 Supplement, and then reseeded in 24-well plates at a density of 2 × 10^5^ cells/well. Primary astrocytes were allowed to grow for 16 h before being infected. Approximately 95% of the cultured cells were identified as astrocytes based on glial fibrillary acidic protein staining positivity, as determined by a previously described immunofluorescence assay [2].

## Microglial cultures

Microglial cells were obtained using a procedure similar to that used for astrocytes, with some modifications. Dissociated brain cells were washed and suspended in DMEM/F-12 supplemented with penicillin-streptomycin, 10% FBS and 10 ng/ml of granulocyte–macrophage colony-stimulating factor (R&D Systems, Minneapolis, MN, USA). The cells were plated in 75-cm^2^ flasks (density, 4 × 10^6^ cells/flask), and the culture medium was changed every 3 days. After 10–11 days of incubation, the microglial cells were detached from the astrocyte monolayer by pipetting. Suspended cells were centrifuged and reseeded in 24-well plates (density, 2 × 10^5^ cells/well). Primary microglial cells were allowed to grow for 16 h before being infected. Approximately 95% of the cultured cells were identified as microglia based on positive staining for CD11b via flow cytometry, as previously described [2].

## Neuron cultures

Neurons were obtained according to a previously described procedure [3], with some modifications. Brain cells were suspended in DMEM/F-12 supplemented with penicillin-streptomycin and B27 supplement (Gibco-BRL), and then plated in 24-well plates (density, 1 × 10^6^ cells/well). Culture medium was changed every 3 days. Primary neurons were allowed to grow for 8 days before being infected.

## Reverse transcription quantitative PCR (RT-qPCR) for brain tissue analysis

Reactions were performed using the following parameters: 2 min at 50°C, 10 min at 95°C, 40 cycles of 15 s at 95°C, and 1 min at 60°C, followed by a dissociation step from 60°C to 95°C to check for gene-specific amplification. Amplification, data acquisition and data analysis were performed in an ABI Prism 7900HT Sequence Detection System (Applied Biosystems, Foster City, CA, USA). The calculated cycle threshold (C_t_) data were normalized to the expression level of the internal control gene (*Actb*) and then analyzed using the 2^-ΔΔCt^ method [4] where ΔΔC_t_  =  ΔC_t_ sample – ΔC_t_ calibrator sample, in which ΔC_t_  =  C_t_ (studied gene) – C_t_ (internal control gene). The internal control gene was selected from three genes (i.e., 18S rRNA, *Gapdh*, *Actb*) using RefFinder [5]. The calibrator sample was the uninfected WT group. When the expression of a target gene (i.e., *Csf3*, *Ifng*) was not detected in the uninfected WT group, the infected WT group was used as the calibrator. A two-way analysis of variance (ANOVA) with post-hoc Tukey’s test was performed to examine the main effects and interaction of the two factors; specifically, the mouse genotype and infection status, and to compare the gene expression levels among the groups.

## DNA extraction, quantitative PCR and T. gondii detection

Following RNA extraction, DNA from the 7-dpi samples was extracted with TRI reagent according to the manufacturer’s protocol to determine the parasite burden in the brain. At 30 dpi, DNA was extracted from the other brain half with phenol–chloroform–isoamyl alcohol (ratio, 25:24:1; Sigma-Aldrich) according to the manufacturer’s protocol. The 25 µl PCR mixtures contained 6.25 µl of Power SYBR™ Green PCR Master Mix (Thermo Fisher Scientific Inc., Waltham, MA, USA), 800 nM of each forward and reverse primer and 50 ng of genomic DNA. The primers used were for the *T. gondii* B1 gene (5′-AAC GGG CGA GTA GCA CCT GAG GAG-3′ and 5′-TGG GTC TAC GTC GAT GGC ATG ACA AC-3′ for forward and reverse, respectively), which is present in all known parasite strains [6]. The thermal cycling protocol was the same as that used for the expression analysis above. Samples were run in duplicate. Parasite numbers per 50 ng of tissue DNA were calculated from a standard curve, which was established from 1 µl of serially diluted *T. gondii* DNA extracted from 1 × 10^5^ parasites, and corresponded to 0.01 to 10,000 parasites per reaction.

# References

1. Fischer HG, Nitzgen B, Germann T, Degitz K, Däubener W, Hadding U. Differentiation driven by granulocyte-macrophage colony-stimulating factor endows microglia with interferon-gamma-independent antigen presentation function. J Neuroimmunol. 1993;42:87–95.

2. Umeda K, Tanaka S, Ihara F, Yamagishi J, Suzuki Y, Nishikawa Y. Transcriptional profiling of Toll-like receptor 2-deficient primary murine brain cells during *Toxoplasma gondii* infection. PLOS ONE. 2017;12:e0187703.

3. Hilgenberg LGW, Smith MA. Preparation of Dissociated Mouse Cortical Neuron Cultures. J Vis Exp JoVE. 2007. doi:10.3791/562.

4. Bonfá G, Benevides L, Souza M do C, Fonseca DM, Mineo TWP, Rossi MA, et al. CCR5 Controls Immune and Metabolic Functions during *Toxoplasma gondii* Infection. PLoS ONE. 2014;9. doi:10.1371/journal.pone.0104736.

5. Xie F, Xiao P, Chen D, Xu L, Zhang B. miRDeepFinder: a miRNA analysis tool for deep sequencing of plant small RNAs. Plant Mol Biol. 2012;80:75–84.

6. Contini C, Seraceni S, Cultrera R, Incorvaia C, Sebastiani A, Picot S. Evaluation of a Real-time PCR-based assay using the lightcycler system for detection of *Toxoplasma gondii* bradyzoite genes in blood specimens from patients with toxoplasmic retinochoroiditis. Int J Parasitol. 2005;35:275–83.
